# The influence of social mobility perception and expectation on residents’ health: the role of subjective well-being and physical exercise

**DOI:** 10.3389/fpubh.2025.1562862

**Published:** 2025-04-02

**Authors:** Xi Liu, Junyi Zheng, Yan Kuang

**Affiliations:** ^1^College of Public Administration, Huazhong University of Science and Technology, Wuhan, China; ^2^School of Physical Education, Huazhong University of Science and Technology, Wuhan, China; ^3^School of Politics and Public Administration, Zhengzhou University, Zhengzhou, China

**Keywords:** social mobility perception, social mobility expectation, self-rated health, subjective well-being, physical exercise

## Abstract

**Background:**

Residents’ Health is crucial for the effective implementation of the “Healthy China 2030” strategy and the sustainable development of society. However, there is still significant room for advancement in academic discussions on ensuring residents’ health.

**Objective:**

This study aims to explore the relationship between social mobility perception and expectation and residents’ self-rated health, and further explore the mediating role of subjective well-being in this, the moderating role of physical exercise, and to provide reference for more targeted development and implementation of residents’ health policies.

**Materials and methods:**

Based on the data collected from 4,372 valid samples in the 2021 Chinese General Social Survey (CGSS), the Bootstrap method was used to test the mechanism of social mobility perception and expectation on residents’ self-rated health.

**Results:**

Both social mobility perception and expectation had significant positive effects on residents’ self-rated health. They indirectly contribute to residents’ self-rated health through the important mediating role of subjective well-being. In addition, physical exercise played an important moderating role in the relationship between social mobility perception and residents’ self-rated health. However, physical exercise did not have a significant moderating effect on the relationship between social mobility expectations and residents’ self-rated health.

**Conclusion:**

This study expands the understanding of the relationship between social mobility and residents’ health in China, providing practical insights for effective strategies to promote residents’ health.

## 1 Introduction

Self-rated health, as a crucial indicator in health research, refers to an individual’s perception and assessment of their overall health status. It serves as a key predictor of life satisfaction, mortality, and overall public health conditions (1–3). For a long time, human health has been a focal concern for governments and organizations worldwide. Numerous actions and measures have been introduced, such as ensuring food security, improving living environments, enhancing public health services, and reducing poverty, all aimed at improving overall health levels (4–7). Entering the 21st century, China’s rapid economic development and continuous improvement in living standards have significantly enhanced public health. Notably, following the release of the Healthy China 2030 blueprint, improving national health literacy has been elevated to a national strategic priority. According to data from the 14th Five-Year Plan for National Health, China’s average life expectancy increased from 76.34 years in 2015 to 77.93 years in 2020, with key health indicators ranking among the top in middle- and high-income countries (8).

However, despite continuous economic growth and rising life expectancy, research has identified a “development paradox,” wherein the public’s self-rated health has not improved proportionally. Moreover, significant disparities exist in health assessments (9, 10). Existing research indicates that self-rated health is influenced by various factors, including personality traits, educational attainment, occupational type, health behaviors, and public health standards (11–15). However, the impact of social stratification and social mobility on health remains to be further explored.

Existing research has primarily focused on a static perspective of social stratification, with particular attention to specific groups such as the older adult and adolescents. Moreover, most studies emphasize the impact of macro-level social mobility, while relatively little attention has been given to individuals’ perceived and expected social mobility at the micro level (16, 17). Even when social mobility perception and expectation were included as boundary conditions in research, the specific mechanisms through which they influence residents’ self-rated health remain unclear. Social mobility not only reflects an individual’s change in social class but also involves the acquisition and loss of related resources and benefits (18). The theory of acculturation provides a theoretical basis for explaining the relationship between perceived and expected social mobility and self-rated health (19). According to the theory of acculturation, the impact of social mobility on health largely depends on the extent to which an individual integrates into a new social class environment. The higher the degree of social integration, the better the health status. When individuals develop an upward social mobility perception, they are more likely to accept the lifestyles of the new social class, establish values compatible with the new class, and construct their own social relationships, thereby increasing social integration and thus self-rated health (20, 21). Downward social mobility perceptions can have a “falling from grace,” on self-rated health (22). When individuals perceive downward social mobility, they may experience feelings of loss and frustration, leading to resistance to the downward cultural adaptation process. This, in turn, can reduce their level of social integration. Moreover, maintaining the lifestyle and social relationships of the original social class can be costly, and value disorientation can lead to increased psychological stress, which negatively affects individuals’ self-rated health.

In addition, this study anticipated that social mobility expectations would also have an impact on self-rated health. Established research has found that social mobility expectations have a greater impact on individuals than social mobility perceptions (23). From a health management perspective, upward social mobility expectations lead individuals to be more open to making changes to their lifestyles and to pay more attention to the maintenance of their own health in order to sustain upward social mobility. According to the falling from grace, when individuals anticipate downward social mobility expectation, it can lead to negative psychological states such as social disorientation or confusion. This, in turn, may result in neglecting health management and even adopting unhealthy lifestyles, such as excessive drinking or overeating, which can deteriorate their health status.

In addition to direct effects, the underlying mechanisms through which social mobility perception and social mobility expectation influence self-rated health need further analysis. At the current stage of social development in China, the public has begun to shift its focus from objective economic indicators such as income and GDP to the improvement of subjective social indicators, such as subjective well-being and sense of gain. Subjective well-being, as an abstract subjective feeling, refers to an individual’s overall perception and evaluation of life quality (24). Existing research has verified that individual health is an important factor influencing subjective well-being (25, 26). However, whether subjective well-being can impact residents’ health still requires further exploration. This study anticipates that subjective well-being will have a positive impact on residents’ health. From a basic medical perspective, research has shown that positive emotions can have beneficial effects on the physiological functions of the heart (27). Conversely, negative emotions can exacerbate symptoms of depression, anxiety, and poor physical health (28). Moreover, the enhancement of subjective well-being can positively impact an individual’s health status through its effects on the autonomic nervous system, immune system, and hormone levels (29). From sociological and psychological perspectives, subjective well-being primarily promotes individuals’ physical and mental health by enhancing economic income, social capital, and health behaviors. Specifically, from an economic income perspective, groups with higher subjective well-being tend to have higher levels of positive emotions (30). This can enhance their work efficiency, leading to higher income levels, which in turn increases their investment in health and benefits their overall health. Secondly, from a social capital perspective, groups with higher subjective well-being are more likely to engage in social activities and are more willing to help others, which helps them build a strong social support network. Through interactions within social networks, individuals can receive more emotional and material support, effectively buffering the negative health impacts caused by stress, anxiety, and other factors. Finally, from a health behavior perspective, subjective well-being can enhance individual health by improving the production and allocation efficiency of healthy behaviors. Specifically, groups with higher subjective well-being are more likely to choose healthier lifestyles (31). They tend to have a more positive attitude toward life and better health awareness, which encourages habits beneficial to health, such as physical exercise and balanced nutrition, while inhibiting unhealthy behaviors like smoking and excessive drinking (32), thereby positively promoting their health.

The impact of social mobility perception and social mobility expectation on self-rated health not only exists directly but may also have an indirect effect through subjective well-being as a psychological variable. Upward social mobility perception leads individuals to feel they have access to more resources, thereby enhancing subjective well-being, which in turn improves self-rated health. In contrast, downward social mobility perception may lead to strong feelings of relative deprivation and frustration (33), reducing subjective well-being and negatively impacting self-rated health. Therefore, subjective well-being acts as a mediator between social mobility perception and self-rated health.

According to the tunnel effect theory, an individual’s welfare utility level depends not only on the resources they currently possess but also on their expectations of future conditions (34). Therefore, when individuals anticipate upward social mobility, they tend to believe they will have more opportunities to access resources (35), and their personal utility level increases accordingly (36). Conversely, downward social mobility expectation leads individuals to perceive that the benefits and resources they currently possess are about to be compromised, which induces negative emotions such as frustration and anxiety. This fosters a pessimistic outlook on the future, thereby reducing their level of subjective well-being. Therefore, social mobility expectation not only directly impacts residents’ self-rated health but also influences it by enhancing their subjective well-being.

Residents’ physical and mental health is the result of the interplay of multiple factors. The interaction between cognitive and behavioral factors can better explain residents’ health. Therefore, after establishing the mediating effect model of the impact of social mobility perception and social mobility expectation on health, this study still needs to further examine the influence of moderating factors in order to more comprehensively explain the multiple pathways for improving residents’ health. Physical exercise is an important behavioral factor influencing health. It refers to the conscious and planned physical activities, such as sports, recreational activities, and leisure, that individuals engage in to promote physical and mental development, enrich their lives, and enhance social interactions (37). Existing research has explored the significant positive impact of physical exercise on individuals across various age groups, including enhancing cognitive function, improving quality of life, boosting emotional and mental health, and improving the functionality of specific populations (38–42). Studies have also shown that individuals with better physical and mental health typically engage in higher levels of physical exercise than those in poorer health (43), and active participation in physical exercise can effectively promote both physical and mental health. Research has confirmed that physical exercise plays an important role in the relationship between socioeconomic status and individual health. The higher the level of participation in physical exercise, the smaller the impact of social status differences on health (44). Based on this, it can be inferred that when the level of participation in physical exercise is higher, the effect of upward social mobility perception and expectation on improving health will be weakened. Because they are more likely to attribute improvements in their health status to the enhancement of their physical exercise levels. Similarly, although downward social mobility perception and expectation may lead to a decline in self-rated health, individuals tend to believe that they can offset the negative health effects brought by downward social mobility through physical exercise, thus preventing a significant decrease in self-rated health. Based on the stress-buffering hypothesis and the conservation of resources theory (45, 46), regular physical exercise can enhance individuals’ stress resistance (47, 48) and improve health status through physiological mechanisms such as endorphin release and improved sleep quality (49).Therefore, individuals who engage in frequent physical exercise may more effectively buffer the resource depletion caused by social mobility perception and expectation, thereby maintaining better self-rated health. Conversely, when the level of participation in physical exercise is low, the effect of upward social mobility perception and upward social mobility expectation on health is greater. Similarly, under the influence of relative deprivation, lower levels of physical exercise will amplify the negative impact of perceived and expected downward social mobility on residents’ health. Therefore, this study posits that physical exercise plays a moderating role in the impact of social mobility perception and expectation on residents’ self-rated health.

In summary, this study attempts to explore the mechanism through which social mobility perception and expectation influence self-rated health at the micro level, which has macro-policy significance, and examines the roles of subjective well-being and physical exercise in this process. Based on the above theoretical derivation and analysis, the research hypotheses proposed in this study are shown in Table 1.

**Table 1:** Summary of research hypotheses.

	Hypothetical content
H1	Social mobility perception has a significant positive impact on self-rated health.
H2	Social mobility expectation has a significant positive impact on self-rated health.
H3	Subjective well-being has a significant positive impact on self-rated health.
H4	Social mobility perception has a significant positive impact on subjective well-being.
H5	Subjective well-being mediates the relationship between social mobility perception and self-rated health.
H6	Social mobility expectation has a significant positive impact on subjective well-being.
H7	Subjective well-being mediates the relationship between social mobility expectation and self-rated health.
H8	Physical exercise moderates the relationship between social mobility perception and self-rated health.
H9	Physical exercise moderates the relationship between social mobility expectation and self-rated health.

**Figure 1:**
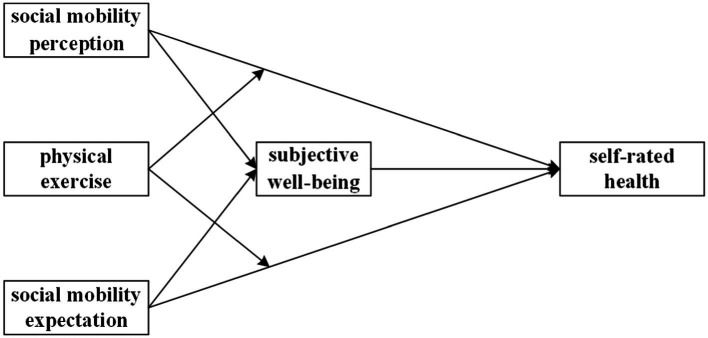
The research model of the study.

The theoretical model constructed in this study is shown in Figure 1.

## 2 Materials and methods

### 2.1 Sample and procedure

The data used in this study comes from the 2021 Chinese General Social Survey (CGSS). The CGSS is a large-scale social survey project organized by the National Survey Research Center of Renmin University of China, conducted annually. It is the longest-running, nationwide, comprehensive, and continuous academic survey project in China. CGSS 2021 adopted a multi-stage stratified PPS random sampling method to select respondents from 29 provinces, municipalities, and autonomous regions in China. Face-to-face interviews were conducted to ensure the most effective representation of various aspects of Chinese society. The survey data in CGSS 2021 includes a total of 8,148 samples; however, not every question was answered completely and accurately by every respondent. This study primarily focuses on variables such as social mobility, self-rated health, and subjective well-being. Based on this, data from CGSS 2021 were filtered, with invalid data such as “do not know,” “not applicable,” “refused to answer,” and missing responses removed. As a result, 4,372 valid samples were obtained for analysis, and the demographic characteristics of the sample are shown in Table 2.

**Table 2:** Sample demographics (*n* = 4,372).

Characteristic	Classification	Frequency	Percentage
Gender	Male	2,045	46.8
	Female	2,327	53.2
Age	18–30	634	14.5
	31–40	665	15.2
	41–50	690	15.8
	51–60	878	20.1
	61–70	870	19.9
	70 years old or above	635	14.5
Annual income	Less than 1,000	897	20.5
	1,000–12,000 (inclusive)	885	20.3
	12,000–35,000 (inclusive)	870	19.9
	35,000–60,000 (inclusive)	929	21.2
	Greater than 60,000	791	18.1
Hukou type	Agricultural	2,485	56.8
	Non-agricultural	1,887	43.2
Education	Junior high school or below	2,611	59.7
	Senior high school	811	18.6
	Associate degree	365	8.3
	Bachelor’s degree	524	12.0
	Master’s degree or above	61	1.4
Marital status	Unmarried	677	15.5
	Married	3,695	84.5

The gender distribution of the valid sample is relatively balanced, with a slightly smaller proportion of males, totaling 2,045 individuals (46.8% of the total), and a slightly larger proportion of females, totaling 2,327 individuals (53.2%). Most respondents are middle-aged and young adults, with an average age of 51.54 years. The youngest respondent is 18 years old, and the oldest is 95 years old. There are 897 respondents with an annual income of less than 1,000, accounting for 20.5%; 885 respondents with an income between 1,000 and 12,000 (inclusive), accounting for 20.3%; 870 respondents with an income between 12,000 and 35,000 (inclusive), accounting for 19.9%; 929 respondents with an income between 35,000 and 60,000 (inclusive), accounting for 21.2%; and 791 respondents with an income greater than 60,000, accounting for 18.1%. The sample includes a higher proportion of rural household registrations, with 2,485 respondents, accounting for 56.8% of the total; while 1,887 respondents have non-rural household registrations, accounting for 43.2%. Among the respondents, 2,611 had education levels of junior high school or below, accounting for 59.7%; 811 had high school education, accounting for 18.6%; 365 had college diplomas, accounting for 8.3%; 524 had bachelor’s degrees, accounting for 12.0%; and 61 had graduate degrees or above, accounting for 1.4%. There are 677 unmarried respondents, accounting for 15.5%, and 3,695 married respondents, accounting for 84.5%.

### 2.2 Measures

#### 2.2.1 Self-rated health

This study uses items from the CGSS 2021 questionnaire that assess residents’ perceptions of their own health to measure self-rated health. The item employs a five-point scale, asking the question, “How would you rate your current physical health status?” The available response options are: “Very unhealthy,” “Somewhat unhealthy,” “Average,” “Somewhat healthy,” and “Very healthy,” which are assigned scores of 1 to 5, respectively.

#### 2.2.2 Social mobility perception and social mobility expectation

This study calculates the data for social mobility perception and social mobility expectation based on three items related to social class in the CGSS 2021 questionnaire. The CGSS 2021 categorizes social class into 10 levels, and respondents are asked to select the social class level they currently belong to, the one they belonged to 10 years ago, and the one they expect to belong to in 10 years. A higher score indicates a stronger identification with a higher social class. This study measures social mobility perception by subtracting the social class identity from 10 years ago from the current social class identity. Social mobility expectation is measured by the difference between the social class identity the respondent expects to belong to in 10 years and their current social class identity. This approach is consistent with the measurement methods used in existing research and helps to reduce systematic measurement errors in individuals’ self-assessments (50, 51).

#### 2.2.3 Subjective well-being

Subjective well-being is generally measured by dividing it into several different levels, allowing respondents to choose the level that best reflects their sense of happiness. Commonly used methods include three-point, four-point, and five-point scales. The CGSS 2021 questionnaire uses a five-point scale to measure subjective well-being. Respondents are asked, “Overall, how would you rate your happiness in life?” The response options are: “Very unhappy,” “Somewhat unhappy,” “Neither happy nor unhappy,” “Somewhat happy,” and “Very happy” with scores assigned as 1 to 5.

#### 2.2.4 Physical exercise

This study measures residents’ physical exercise based on the item in the CGSS 2021 questionnaire: “In the past year, have you frequently engaged in physical exercise during your leisure time?” The response options include: “Every day,” “Several times a week,” “Several times a month,” “Once a year or less,” and “Never.” Following existing research, this study constructs a binary dummy variable for physical exercise based on the respondents’ answers (52, 53). In this study, “0 = did not engage in physical exercise, 1 = engaged in physical exercise.”

#### 2.2.5 Control variables

Previous research has found significant individual differences in self-rated health and subjective well-being, with variations based on factors such as gender, age, ethnicity, and socio-economic status (54–56). Therefore, this study includes gender, age, personal income, household registration type, education level, and marital status as control variables in our analysis. Gender is a binary variable, with 1 representing male and 2 representing female. Hukou type and marital status are operationalized as binary variables. Hukou type is divided into agricultural hukou (1) and non-agricultural hukou (2), while marital status is divided into unmarried (1) and married (2). Education level is operationalized as a five-category dummy variable (junior high school or below, high school, junior college, bachelor’s degree, and graduate school or above). Age is a continuous variable, ranging from 18 to 95 years. Personal income is treated as a continuous variable, with a range of values from 1 to 5. Due to the significant skewness of the personal income variable, the absolute household income variable is log-transformed and included in the regression model for a more intuitive comparison of the regression coefficients.

### 2.3 Analysis

In this study, descriptive statistical analysis is used to describe the basic characteristics of the sample. In addition, this study employs the conditional process analysis method proposed by Hayes (57) to test the mediating role of subjective well-being and the moderating role of physical exercise. The simple mediation model (Model 4) in SPSS macro estimates the mediating role of subjective well-being in the relationship between social mobility perception and expectation and residents’ health. The bootstrap method was used to estimate the 95% confidence intervals of 4,372 random samples and test the significance of the mediation effect. If the 95% confidence interval does not include 0, it indicates statistical significance. Subsequently, considering the relevant control variables, the moderated mediation model was tested using Model 59 in the SPSS macro. The model formula is as follows:

$$Y=\lambda_{0}+\lambda_{1}X+\lambda_{2}C_{1}$$

$$M=\beta_{0}+\beta_{1}X+\beta_{2}M_{i}+\beta_{3}C_{2}$$

$$Y=\gamma_{0}+\gamma_{1}X+\gamma_{2}M_{i}+\gamma_{3}W+\gamma_{4}XW+\gamma_{5}M_{i}W+\gamma_{6}C_{3}$$

Where X represents the independent variable, Y represents the dependent variable, M represents the mediator variable, and W represents the moderator variable. C1, C2, and C3 represent the control variables. To test the moderating effect, the regression equation included the moderating variable W, as well as the interaction terms between X and W, and M and W. The parameter tests for $\gamma_{4}$ and $\gamma_{5}$ help determine whether W significantly moderates the direct effect of the independent variable X on the dependent variable Y, as well as the effect of the mediator variable M. Additionally, this study conducted a simple slope analysis, which involved substituting the values of the moderating variable for non-participants in physical exercise (*W* = 0) and participants in physical exercise (*W* = 1) into the equation. This method illustrates the changes in the direct, indirect, and total effects under different values of the moderator variable. The specific statistical analysis was conducted using SPSS 27.0 and the PROCESS macro (57).

Since the measurement of the core variables in this study is based on subjective judgment items, the data generated from self-reports may be subject to common method bias. The results show that the variance explained by the largest factor is 30.93% (below the 40% threshold), indicating that there is no significant common method bias in the data of this study.

## 3 Results

### 3.1 Descriptive statistics and correlation analysis

This study used SPSS 27.0 software to perform descriptive statistical analysis and correlation tests on the main variables. The mean, standard deviation, maximum value, minimum value, and correlation analysis results of the variables are shown in Table 3. The results indicate that perceived social mobility is significantly positively correlated with self-rated health (*r* = 0.146, *p* < 0.001), and social mobility expectation are also significantly positively correlated with self-rated health (*r* = 0.175, *p* < 0.001). At the same time, perceived social mobility is significantly positively correlated with subjective well-being (*r* = 0.150, *p* < 0.001), social mobility expectation is significantly positively correlated with subjective well-being (*r* = 0.059, *p* < 0.001), and subjective well-being is significantly positively correlated with self-rated health (*r* = 0.241, *p* < 0.001). These results provide preliminary support for hypotheses 1, 2, 3, 4, and 6. In addition, physical exercise is significantly positively correlated with perceived social mobility (*r* = 0.036, *p* < 0.01), social mobility expectation (*r* = 0.097, *p* < 0.001), subjective well-being (*r* = 0.113, *p* < 0.001), and self-rated health (*r* = 0.194, *p* < 0.001).

**Table 3:** Means, standard deviations, and correlations among study variables (*n* = 4,372).

	Mean	SD	Min	Max	1	2	3	4
SMP	0.612	1.607	−9.00	9.00				
SME	0.807	1.510	−8.00	9.00	0.101^***^			
SWB	3.980	0.817	1	5	0.150^***^	0.059^***^		
PE	0.670	0.470	0	1	0.036^**^	0.097^***^	0.113^***^	
SRH	3.500	1.079	1	5	0.146^***^	0.175^***^	0.241^***^	0.194^***^

SMP, social mobility perception; SME, social mobility expectation; SWB, subjective well-being; PE, physical exercise; SRH, self-rated health. ^**^p < 0.01, ^***^p < 0.001.

### 3.2 Hypothesis testing

#### 3.2.1 Direct and mediating effects

This study used SPSS 27.0 statistical software and applied linear regression to conduct a simple effect analysis of the impact of perceived social mobility and social mobility expectation on self-rated health. The results are shown in Tables 4, 5. Model 2 in Table 4 presents the linear regression results, while Table 5 shows the main effects of perceived social mobility and social mobility expectation on self-rated health. The analysis results indicate that, after controlling for other variables, perceived social mobility has a significant positive impact on residents’ self-rated health (*r* = 0.073, *p* < 0.001), with a confidence interval of [0.055, 0.092] that does not include 0, thus reaching statistical significance. Hypothesis 1 is confirmed, meaning that the higher the perceived social mobility, the higher the residents’ self-rated health. The positive impact of social mobility expectation on residents’ self-rated health is significant (*r* = 0.045, *p* < 0.001), with a confidence interval of [0.024, 0.066] that does not include 0. This supports Hypothesis 2, meaning residents with higher social mobility expectation tend to have higher self-rated health. In terms of standardized coefficients, the effect of perceived social mobility on residents’ health is greater than the effect of social mobility expectation.

**Table 4:** Mediating effect of subjective well-being (*n* = 4,372).

Variables	Self-rated health				Subjective well-being	
	Model 1	Model 2	Model 3	Model 4	Model 5	Model 6
Age	−0.345^***^	−0.342^***^	−0.356^***^	−0.348^***^	0.092^***^	0.106^***^
Gender	−0.031^**^	−0.035^**^	−0.034^**^	−0.034^**^	0.006	0.007
Education	0.037^**^	0.009	0.007	0.008	0.125^***^	0.129^***^
Income	0.076^***^	0.064^***^	0.064^***^	0.067^***^	0.028^*^	0.036^**^
Hukou type	0.038^**^	0.041^***^	0.039^**^	0.036^**^	0.026	0.020
Marital status	−0.003	−0.005	−0.006	−0.006	0.020	0.020
SMP	–	0.071^***^	0.073^***^	–	0.156^***^	–
SME	–	0.039^***^	–	0.043^***^	–	0.083^***^
SWB	–	0.227^***^	0.230^***^	0.238^***^	–	–
R^2^	0.147	0.210	0.209	0.205	0.041	0.023
F	124.960	128.912	143.899	140.847	26.354	14.543

SMP, social mobility perception; SME, social mobility expectation; SWB, subjective well-being. ^*^p < 0.05, ^**^p < 0.01, ^***^p < 0.001.

**Table 5:** Decomposition of total, direct, and mediating effects (*n* = 4,372).

	SWB	Effect	SE	t	LLCI	ULCI
SMP → SRH	Total effect	0.073	0.009	7.777^***^	0.055	0.092
	Direct effect	0.049	0.009	5.327^***^	0.031	0.067
		*Effect*	*BootSE*		*BootLLCI*	*BootULCI*
	Mediating effect	0.024	0.003		0.018	0.030
SME → SRH	Total effect	0.045	0.011	4.220^***^	0.024	0.066
	Direct effect	0.031	0.010	2.989^***^	0.011	0.051
		*Effect*	*BootSE*		*BootLLCI*	*BootULCI*
	Mediating effect	0.014	0.003		0.008	0.021

SMP, social mobility perception; SME, social mobility expectation; SWB, subjective well-being; SRH, self-rated health. ^***^p < 0.001.

After controlling for variables such as gender, age, education level, personal income, household registration type, and marital status, this study used SPSS macro model 4 to test the mediation model. The test results are presented in Models 3 to 6 in Table 4. From Models 3 and 5, it can be seen that perceived social mobility has a significant positive impact on both self-rated health and subjective well-being (*r* = 0.073, *p* < 0.001; *r* = 0.156, *p* < 0.001), confirming Hypotheses 1 and 4. Additionally, subjective well-being also has a significant positive impact on residents’ self-rated health (*r* = 0.230, *p* < 0.001), thus supporting Hypothesis 3. As shown in Table 5, the mediating effect of residents’ subjective well-being between perceived social mobility and self-rated health is 0.024, with a 95% confidence interval of [0.018, 0.030]. The direct effect of perceived social mobility on residents’ subjective well-being is 0.049, with a confidence interval of [0.031, 0.067]. Both confidence intervals do not include 0, indicating that the effects are statistically significant. Therefore, it can be concluded that subjective well-being mediates the relationship between perceived social mobility and residents’ self-rated health. Perceived social mobility can enhance residents’ self-rated health through subjective well-being, thus supporting Hypothesis 5.

As shown in Models 4 and 6 of Table 4, both social mobility expectation and subjective well-being have a significant positive impact on residents’ self-rated health (*r* = 0.043, *p* < 0.001; *r* = 0.238, *p* < 0.001). Additionally, social mobility expectation has a significant positive impact on subjective well-being (*r* = 0.083, *p* < 0.001), thereby supporting Hypothesis 6. The mediation effect of subjective well-being between social mobility expectation and self-rated health is confirmed (see Table 5). The mediation effect of subjective well-being is 0.014, with a 95% confidence interval of [0.008, 0.021]. The direct effect of social mobility expectation on self-rated health is 0.031, with a confidence interval of [0.011, 0.051]. Both confidence intervals do not include 0, further confirming that subjective well-being mediates the relationship between social mobility expectation and self-rated health. The increase in social mobility expectation leads to an improvement in subjective well-being, which in turn affects the enhancement of self-rated health. This confirms the validation of Hypothesis 7.

#### 3.2.2 Moderating effects

This study follows Hayes’s (57) proposed moderated mediation analysis model, using Model 5 in the PROCESS macro and the Bootstrap method to test the moderating effect of physical exercise participation, with 5,000 bootstrap samples. The data analysis results are presented in Table 6. First, the interaction term between perceived social mobility and physical exercise has a significant negative effect on self-rated health (*r* = −0.041, *p* < 0.01), indicating that physical exercise moderates the relationship between perceived social mobility and self-rated health. This supports the validation of Hypothesis 8. However, the interaction term between social mobility expectation and physical exercise is not significantly related to self-rated health (*r* = −0.031, ns), indicating that physical exercise does not have a significant moderating effect on the relationship between social mobility expectation and self-rated health. Therefore, Hypothesis 9 is not supported. As residents engage in physical exercise activities, the positive effect of perceived social mobility on self-rated health gradually weakens, while the effect of social mobility expectation on self-rated health does not show a significant change.

**Table 6:** Moderating effect of physical exercise.

Variables	Self-rated Health			
	Coeff	SE	Coeff	SE
Constant	3.174	0.130	3.115	0.133
SMP	0.074^***^	0.015	–	–
SME	–	–	0.050^***^	0.017
SWB	0.296^***^	0.018	0.307^***^	0.018
PE	0.168^***^	0.035	0.164^***^	0.037
SMP × PE	−0.041^**^	0.019	–	–
SME × PE	–	–	−0.031	0.021
Age	−0.022^***^	0.001	−0.021^***^	0.001
Gender	−0.074^**^	0.030	−0.072^**^	0.030
Education	−0.003	0.018	−0.003	0.018
Income	0.063^***^	0.015	0.065^***^	0.015
Hukou type	0.064^*^	0.034	0.059^*^	0.034
Marital status	−0.024	0.048	−0.024	0.048
R^2^	0.213	–	0.209	–

SMP, social mobility perception; SME, social mobility expectation; SWB, subjective well-being. ^*^p < 0.05, ^**^p < 0.01, ^***^p < 0.001.

**Figure 2:**
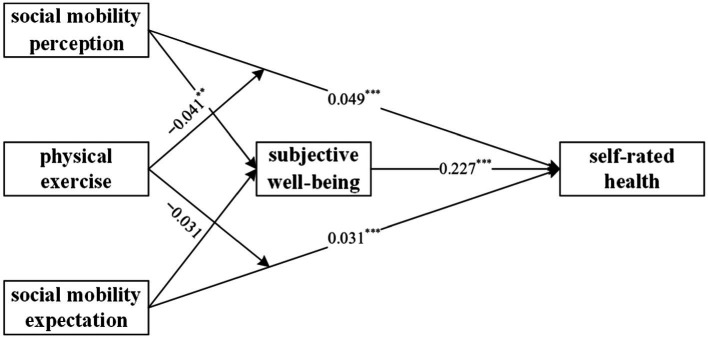
Mechanisms of social mobility perception and expectation on self-rated health.

**Figure 3:**
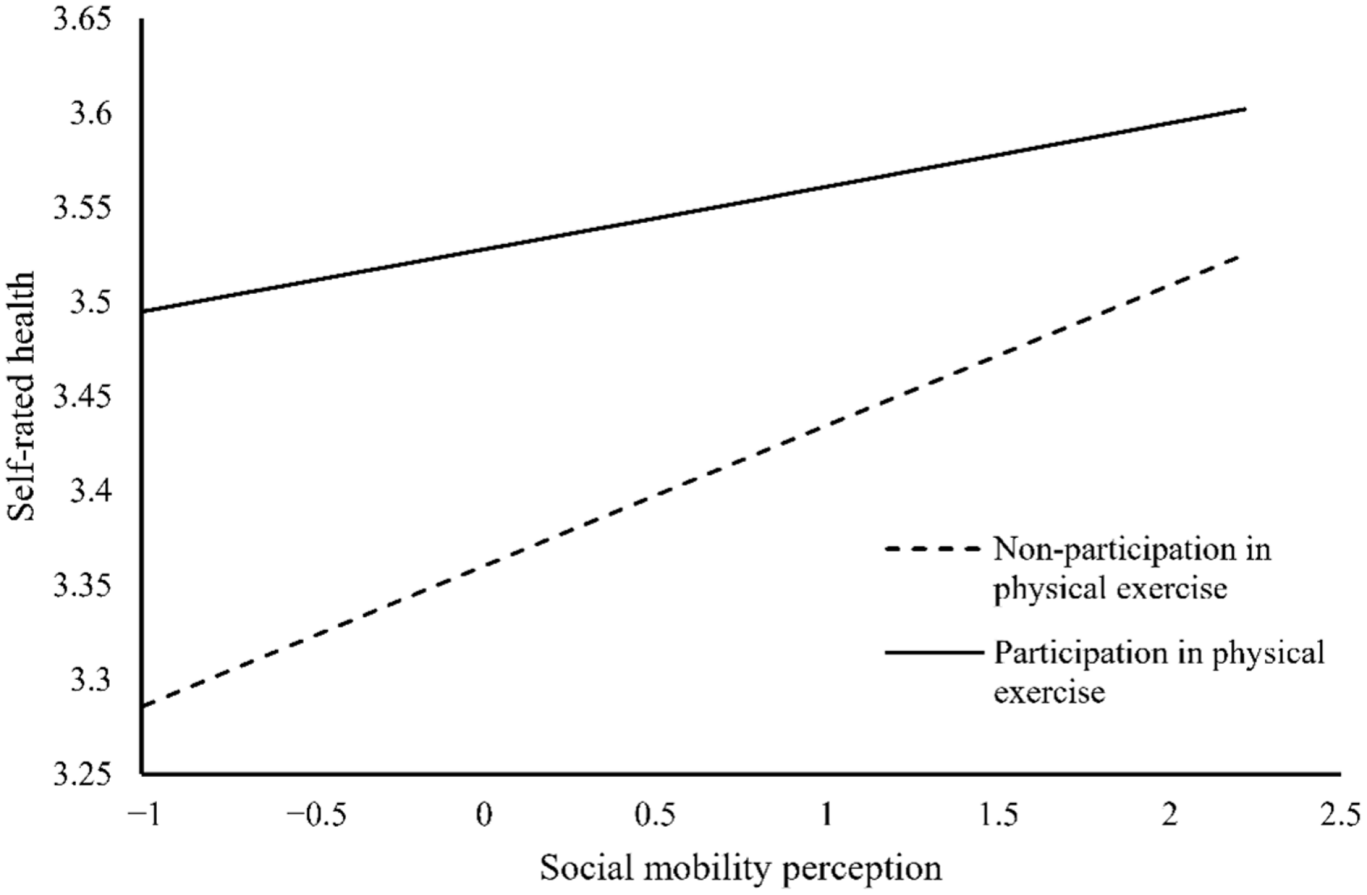
The moderating effect of physical exercise.

Overall, the results of this study, as shown in Figure 2, indicate that both social mobility perception and social mobility expectation have a significant positive impact on subjective well-being and self-rated health. Furthermore, subjective well-being also has a significant positive effect on self-rated health. There are mediating effects of subjective well-being between social mobility perception and self-rated health, as well as between social mobility expectation and self-rated health. Regarding the moderating effect, physical exercise moderates the relationship between social mobility perception and self-rated health, but it does not significantly moderate the relationship between social mobility expectation and self-rated health. Additionally, this study also conducted a simple slope analysis to illustrate the moderating role of physical exercise between perceived social mobility and self-rated health, as shown in Figure 3. Regardless of whether the respondents engage in physical exercise, perceived social mobility significantly and positively predicts self-rated health. However, for individuals who do not engage in physical exercise (*W* = 1), the impact of perceived social mobility on self-rated health is more significant. This result suggests that physical exercise moderates the relationship between perceived social mobility and self-rated health. Specifically, as individuals engage in physical exercise, the impact of perceived social mobility on self-rated health decreases.

## 4 Discussion

This study uses data from the CGSS 2021 to examine the underlying mechanisms and boundary conditions between perceived and expected social mobility and residents’ self-rated health. Through empirical data analysis, this study aims to explain under what conditions perceived and expected social mobility are related to residents’ self-rated health. The relevant discussion and insights are as follows:

First, the study results indicate that perceived and expected social mobility positively influence residents’ self-rated health, as anticipated in this research. Previous research has primarily focused on the impact of socioeconomic status on individual cognition (58). Still, empirical studies examining the effects of perceived and expected social mobility on individual health need further development. Similarly, existing studies have mainly explored the impact of objective economic conditions on health (59, 60), while research on the predictors of self-rated health from an individual’s subjective perspective remains relatively scarce. From a theoretical perspective, the results of this study show that personal income, as a control variable, has a greater impact on residents’ health than other control variables. This is consistent with findings from previous research (61). Furthermore, this study focuses on the impact of individuals’ perception of social mobility on residents’ health. It finds that both perceived and expected social mobility significantly affect residents’ self-rated health. It can be seen that subjective socioeconomic status significantly predicts self-rated health, which is consistent with the conclusions of existing research (62). In addition, the study also found that the effect of social mobility perception on residents’ health is greater than that of social mobility expectation. Therefore, this study provides new insights into the relationship between social mobility and individual health. From a policy perspective, improving residents’ health should not be limited to enhancing personal income as a single economic channel. Rather, attention should be given to a more comprehensive approach that addresses social status as a broader and more integrated factor. The government should build a well-established social mobility mechanism, expand opportunities for social mobility, and promote residents’ social mobility. At the same time, it should optimize the distribution of resources and opportunities to create a favorable social environment. This will allow the positive effects of social mobility on public health to be fully realized.

Second, to better understand how perceived and expected social mobility influence residents’ self-rated health, this study further explores the underlying mechanisms linking perceived and expected social mobility to self-rated health. From a theoretical perspective, the results of this study, based on the 2021 CGSS data, show that subjective well-being partially mediates the relationship between perceived and expected social mobility and residents’ self-rated health. This suggests that subjective well-being is an important mechanism linking social mobility to self-rated health. This study shows that upward social mobility perception and expectation can enhance an individual’s subjective well-being, which in turn is positively correlated with their self-rated health. Therefore, this study validates the important role of individual perception in promoting subjective well-being, which is consistent with previous research (63, 64). From a policy perspective, in addition to improving income levels to enhance residents’ economic conditions, efforts should also be made to comprehensively enhance public subjective well-being through changes in development concepts and the effective implementation of livelihood policies. This will strengthen the role of subjective well-being as a bridge between social mobility perception and expectation and residents’ health.

Third, this study explores the boundary conditions under which perceived and expected social mobility influence residents’ self-rated health. The results show that physical exercise plays a positive moderating role in the relationship between perceived social mobility and residents’ self-rated health, while its moderating effect between social mobility expectation and residents’ health is not significant. It is possible that social mobility perception typically reflects an individual’s immediate feelings about changes in their current social status, which can directly influence psychological stress and physical health. As a stress-relief mechanism, physical exercise may buffer the negative impact of social mobility perception on self-rated health by improving mental well-being (e.g., reducing anxiety and depression) (65). However, social mobility expectation is more related to an individual’s long-term aspirations and planning for the future. This expectation may rely more on psychological resilience and long-term behavioral patterns rather than immediate health behaviors (such as physical exercise). Existing research has found that although individual perception can affect health, self-rated health is also influenced by individual behaviors (15, 66). Existing literature has examined the moderating role of various behavioral variables in the relationship between individual perception and health, such as healthy eating behaviors, mindfulness relaxation, smoking, and others (67, 68). From a theoretical perspective, this study, considering the importance of physical exercise for individual health, explores the moderating role of physical exercise in the relationship between social mobility perception and expectation and residents’ self-rated health. This helps to provide a clearer understanding of the pathway mechanisms through which social mobility perception influence residents’ health. From a policy perspective, efforts should be made to fully promote the national fitness strategy. This should involve tailoring the approach to local conditions and residents’ needs, accelerating the construction of fitness venues, and supporting facilities. At the same time, there should be active development of grassroots national fitness organizations and strengthening the construction of the sports social organization system. In addition, it is essential to leverage online platforms to promote scientific and diverse methods of physical exercise, thereby enhancing the overall level of physical activity and ensuring public health.

### 4.1 Limitations and future research

This study has several limitations. First, the measurement of physical exercise in this research was based on a single item, which is relatively broad and may not fully capture the complexity of individuals’ exercise behaviors. In the future, it is necessary to adopt a multidimensional perspective, using various indicators such as types of exercise, duration, and frequency, to comprehensively measure physical exercise. This would further enhance the reliability of the research findings.

Second, this study used cross-sectional data to test the hypotheses, which cannot reflect the causal relationship between social mobility perception and expectation and residents’ self-rated health. Future research could employ longitudinal or tracking data collection methods to investigate the causal mechanisms between these variables.

## 5 Conclusion

In the context of the “Healthy China 2030” strategy, this study, based on the CGSS 2021 data, proposes a moderated mediation model to reveal whether and how social mobility perception and expectation influence residents’ self-rated health. The empirical results indicate that both social mobility perception and expectation have a significant positive impact on residents’ self-rated health, with social mobility perception having a greater effect on residents’ health than social mobility expectation. Subjective well-being plays a mediating role in the relationship between social mobility perception and expectation and residents’ self-rated health. In addition, physical exercise significantly and positively moderates the relationship between social mobility perception and residents’ self-rated health, while its moderating effect on the relationship between social mobility expectation and residents’ health is not significant. These findings provide valuable insights into how to expand social mobility opportunities and improve residents’ social status, thereby enhancing their sense of happiness and well-being. Additionally, they highlight the importance of increasing physical exercise levels within a reasonable range to effectively safeguard residents’ health. Future research could further explore the differences in effects across different groups and investigate other potential mediating and moderating variables (such as social support, economic stability, and environmental factors), in order to provide more comprehensive theoretical support for the development of public health policies.

## Data Availability

The original contributions presented in the study are included in the article/supplementary material, further inquiries can be directed to the corresponding author.
